# The genome sequence of the Single-dotted Wave, *Idaea dimidiata *(Hufnagel, 1767)

**DOI:** 10.12688/wellcomeopenres.20185.1

**Published:** 2023-12-05

**Authors:** Ian Sims, David C. Lees, Dominic Phillips, Natural History Museum Genome Acquisition Lab, Darwin Tree of Life Barcoding collective, Wellcome Sanger Institute Tree of Life programme, Wellcome Sanger Institute Scientific Operations: DNA Pipelines collective, Tree of Life Core Informatics collective, Darwin Tree of Life Consortium

**Affiliations:** 1 Syngenta International Research Station, Jealott’s Hill, England, UK; 2 Natural History Museum, London, England, UK

**Keywords:** Idaea dimidiata, Single-dotted Wave, genome sequence, chromosomal, Lepidoptera

## Abstract

We present a genome assembly from an individual male *Idaea dimidiata* (the Single-dotted Wave; Arthropoda; Insecta; Lepidoptera; Geometridae). The genome sequence is 422.6 megabases in span. Most of the assembly is scaffolded into 20 chromosomal pseudomolecules, including the Z sex chromosome. The mitochondrial genome has also been assembled and is 17.04 kilobases in length. Gene annotation of this assembly on Ensembl identified 18,351 protein coding genes.

## Species taxonomy

Eukaryota; Metazoa; Eumetazoa; Bilateria; Protostomia; Ecdysozoa; Panarthropoda; Arthropoda; Mandibulata; Pancrustacea; Hexapoda; Insecta; Dicondylia; Pterygota; Neoptera; Endopterygota; Amphiesmenoptera; Lepidoptera; Glossata; Neolepidoptera; Heteroneura; Ditrysia; Obtectomera; Geometroidea; Geometridae; Sterrhinae; *Idaea*; *Idaea dimidiata* (Hufnagel, 1767) (NCBI:txid572776).

## Background

The Single-dotted Wave ( *Idaea dimidiata*) is a Geometrid moth in the Sterrhinae sub-family, with a wingspan of 9–11 mm *. I. dimidiata* is characterised by dark marks distal to the dorsal half of the third forewing line and straw-white colouration on the forewings ( Lewis, 2023; Waring *et al.*, 2017).

*Idaea dimidiata* is a univoltine species, on the wing from June to August, overwintering from the end of August to May as a larva, pupating in plant debris. It can be found visiting flowers between dawn and dusk when in its flight season ( Waring *et al.*, 2017). The wild larvae of *I. dimidiata* feed on the flowers of hedge bedstraw, cow parsley and burnet-saxifrage ( Waring *et al.*, 2017), but in captivity, may feed from herbaceous plants.

A common resident and well distributed throughout England, Ireland, Wales and the Isle of Man, it is local in southern Scotland and is rarely recorded further northwards. Both the distribution and occurrences of *I. dimidiata* have increased significantly in Britain since 1970, possibly as it is found in a wide range of habitats from woodland to marshes and fens ( Randle *et al.*, 2019). Globally, *I. dimidiata* occurs in the Nearctic and western Palearctic, widespread throughout central Europe. Outside of Europe, its range extends from Turkey to central Asia ( GBIF Secretariat, 2023).

A study conducted on Irish Lepidoptera showed that *I. dimidiata* was able to significantly alter its phenology over a period of 35 years in response to increased temperatures. The full genome of *Idaea dimidiata* could help provide insights into how some moth species can alter their phenology at more significant rates than others in response to climate change ( O’Neill *et al.*, 2012).

The genome of *I. dimidiata* was sequenced as part of the Darwin Tree of Life Project, a collaborative effort to sequence all named eukaryotic species in the Atlantic Archipelago of Britain and Ireland. Here we present a chromosomally complete genome sequence for *I. dimidiata*, based on one specimen from Hartslock Woods, Oxfordshire.

## Genome sequence report

**Figure 1.**
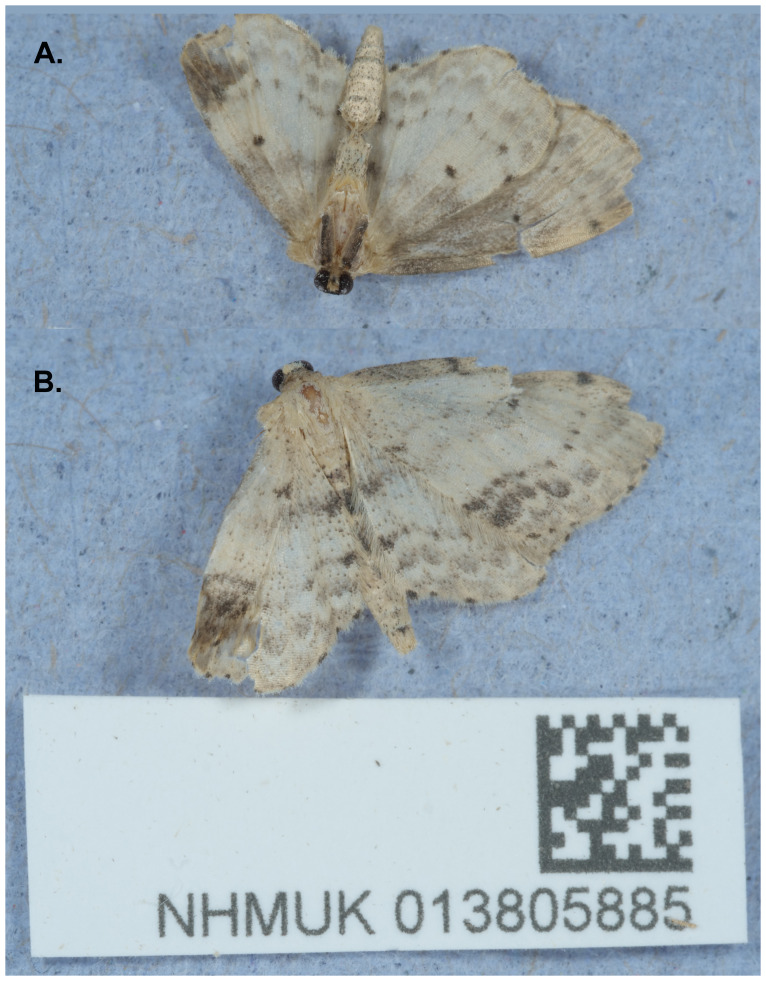
Photographs of the *Idaea dimidiata* (ilIdaDimi2) specimen used for genome sequencing. **A**. Ventral view, **B**. Dorsal view.

The genome was sequenced from one male *Idaea dimidiata* ( Figure 1) collected from Hartslock Nature Reserve, England (51.51, –1.11). A total of 64-fold coverage in Pacific Biosciences single-molecule HiFi long reads was generated. Primary assembly contigs were scaffolded with chromosome conformation Hi-C data. Manual assembly curation corrected 5 missing joins or mis-joins and removed one haplotypic duplication, reducing the assembly length by 1.49% and the scaffold number by 2.7%.

**Figure 2.**
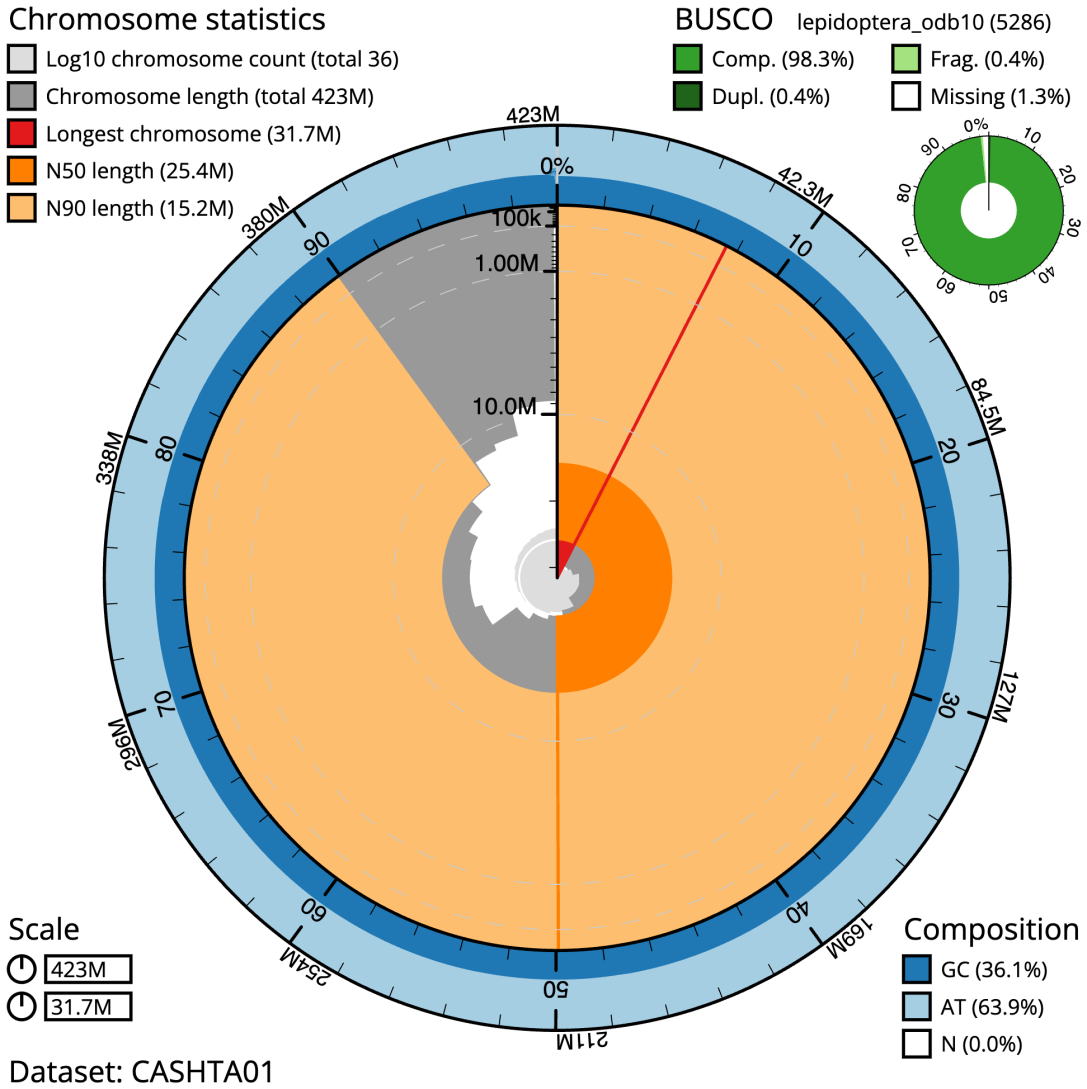
Genome assembly of *Idaea dimidiata*, ilIdaDimi2.1: metrics. The BlobToolKit Snailplot shows N50 metrics and BUSCO gene completeness. The main plot is divided into 1,000 size-ordered bins around the circumference with each bin representing 0.1% of the 422,592,496 bp assembly. The distribution of scaffold lengths is shown in dark grey with the plot radius scaled to the longest scaffold present in the assembly (31,731,116 bp, shown in red). Orange and pale-orange arcs show the N50 and N90 scaffold lengths (25,418,079 and 15,169,836 bp), respectively. The pale grey spiral shows the cumulative scaffold count on a log scale with white scale lines showing successive orders of magnitude. The blue and pale-blue area around the outside of the plot shows the distribution of GC, AT and N percentages in the same bins as the inner plot. A summary of complete, fragmented, duplicated and missing BUSCO genes in the lepidoptera_odb10 set is shown in the top right. An interactive version of this figure is available at https://blobtoolkit.genomehubs.org/view/ilIdaDimi2.1/dataset/CASHTA01/snail.

**Figure 3.**
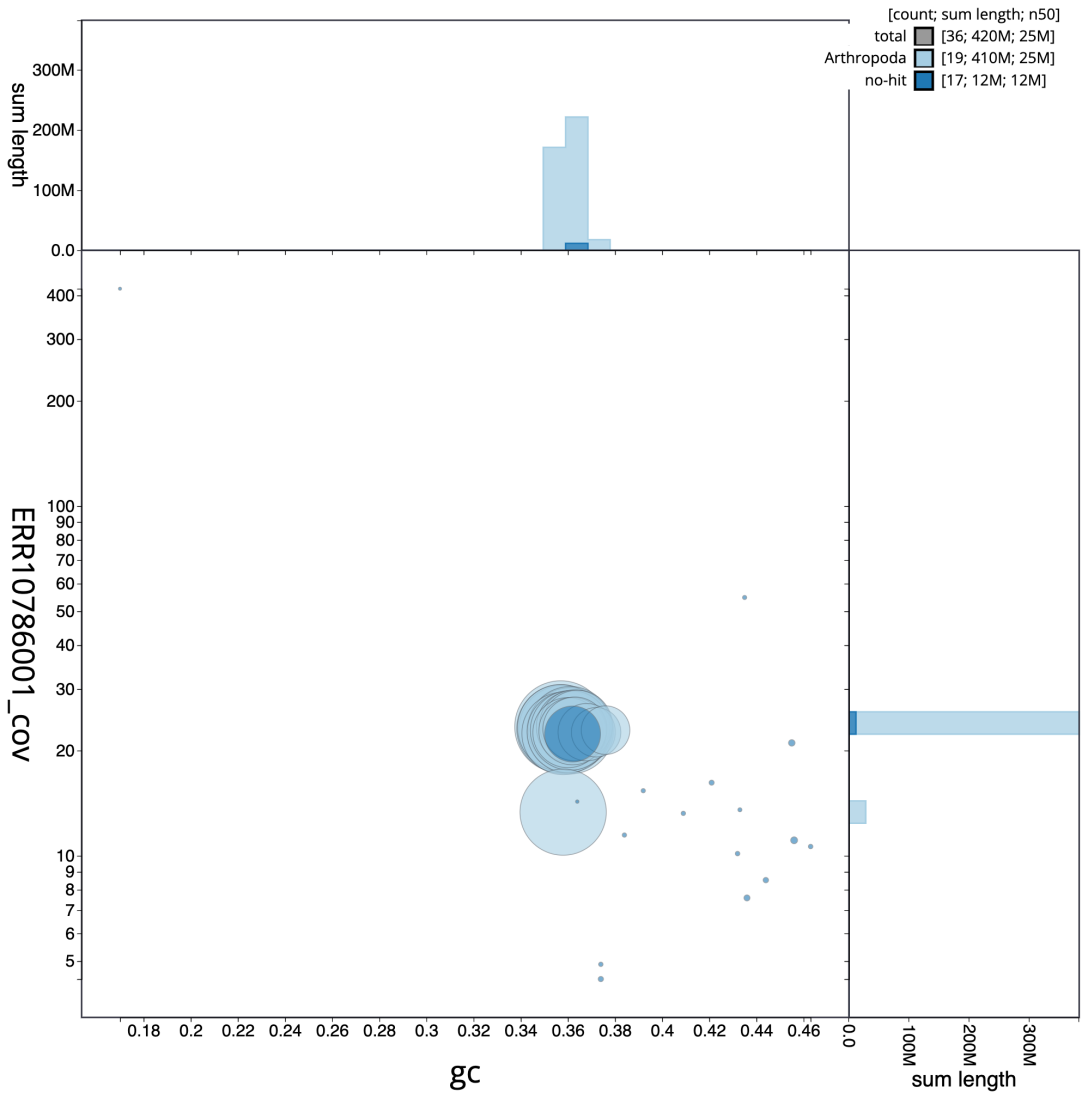
Genome assembly of *Idaea dimidiata*, ilIdaDimi2.1: BlobToolKit GC-coverage plot. Scaffolds are coloured by phylum. Circles are sized in proportion to scaffold length. Histograms show the distribution of scaffold length sum along each axis. An interactive version of this figure is available at https://blobtoolkit.genomehubs.org/view/ilIdaDimi2.1/dataset/CASHTA01/blob.

**Figure 4.**
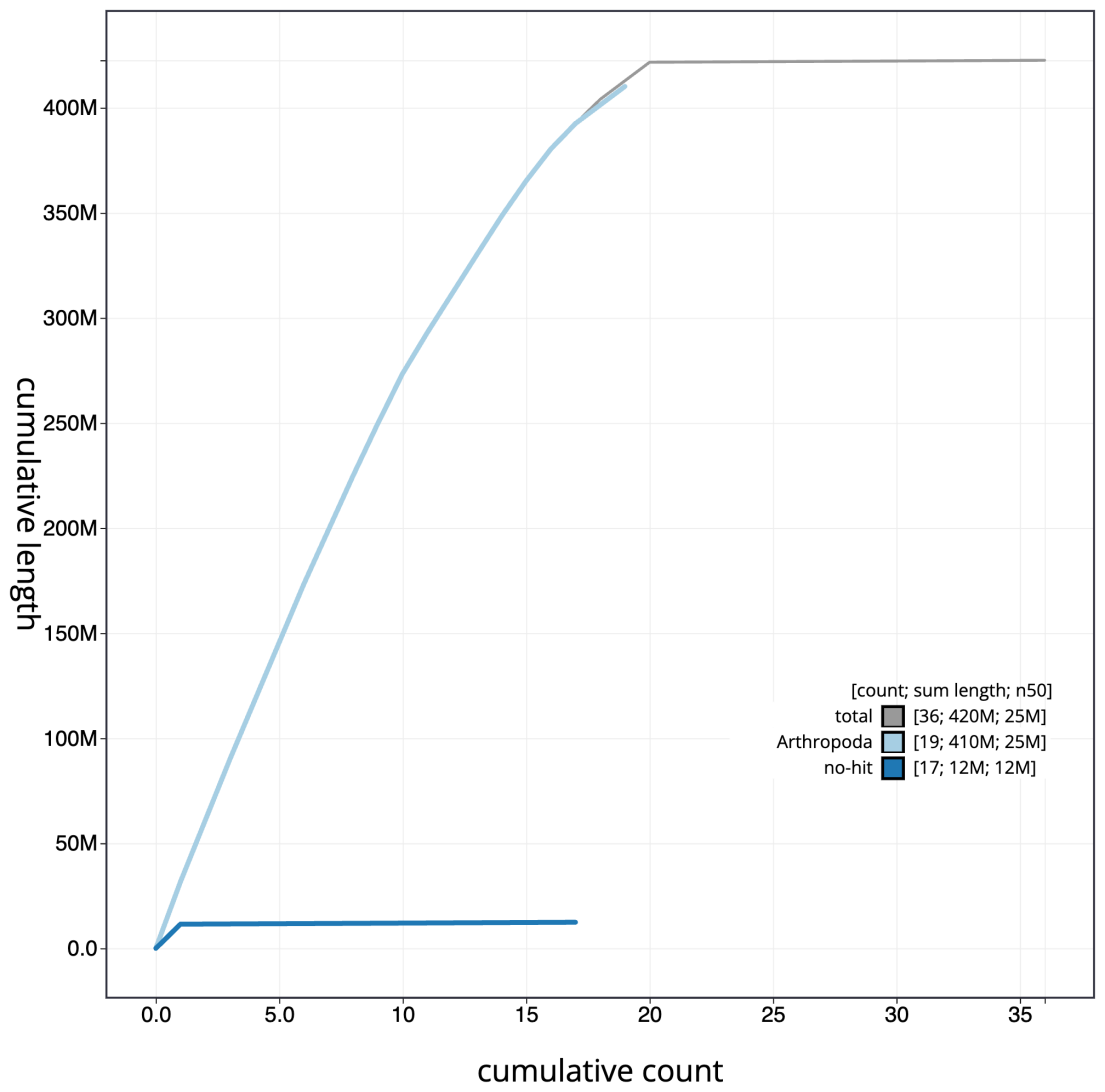
Genome assembly of *Idaea dimidiata*, ilIdaDimi2.1: BlobToolKit cumulative sequence plot. The grey line shows cumulative length for all scaffolds. Coloured lines show cumulative lengths of scaffolds assigned to each phylum using the buscogenes taxrule. An interactive version of this figure is available at https://blobtoolkit.genomehubs.org/view/ilIdaDimi2.1/dataset/CASHTA01/cumulative.

**Figure 5.**
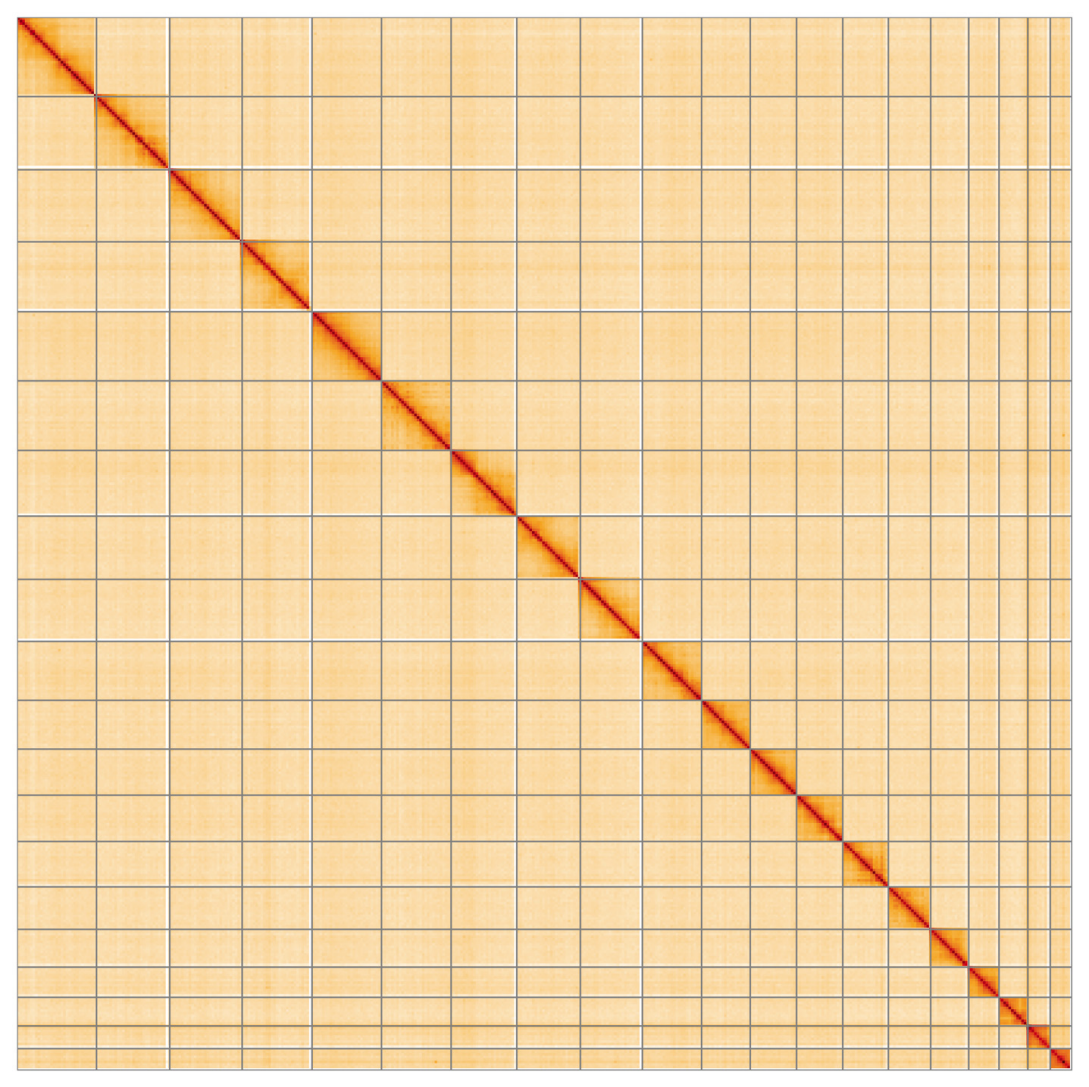
Genome assembly of *Idaea dimidiata*, ilIdaDimi2.1: Hi-C contact map of the ilIdaDimi2.1 assembly, visualised using HiGlass. Chromosomes are shown in order of size from left to right and top to bottom. An interactive version of this figure may be viewed at https://genome-note-higlass.tol.sanger.ac.uk/l/?d=Qnv4yBMZRHaJ-Tq_i8bcnw.

The final assembly has a total length of 422.6 Mb in 35 sequence scaffolds with a scaffold N50 of 25.4 Mb ( Table 1). The snailplot in Figure 2 provides a summary of the assembly statistics, while the distribution of assembly scaffolds on GC proportion and coverage is shown in Figure 3. The cumulative assembly plot in Figure 4 shows curves for subsets of scaffolds assigned to different phyla. Most (99.78%) of the assembly sequence was assigned to 20 chromosomal-level scaffolds, representing 19 autosomes and the Z sex chromosome. Chromosome-scale scaffolds confirmed by the Hi-C data are named in order of size ( Figure 5; Table 2). While not fully phased, the assembly deposited is of one haplotype. Contigs corresponding to the second haplotype have also been deposited. The mitochondrial genome was also assembled and can be found as a contig within the multifasta file of the genome submission.

**Table 1.** Genome data for *Idaea dimidiata*, ilIdaDimi2.1.

Project accession data		
Assembly identifier	ilIdaDimi2.1	
Assembly release date	2023-05-10	
Species	*Idaea dimidiata*	
Specimen	ilIdaDimi2	
NCBI taxonomy ID	572776	
BioProject	PRJEB58928	
BioSample ID	SAMEA111458021	
Isolate information	ilIdaDimi2, male: head and thorax (DNA sequencing and Hi-C scaffolding), abdomen (RNA sequencing)	
Assembly metrics *		*Benchmark*
Consensus quality (QV)	66.5	*≥ 50*
*k*-mer completeness	100%	*≥ 95%*
BUSCO **	C:98.3%[S:97.8%,D:0.4%],F:0.4%, M:1.3%,n:5,286	*C ≥ 95%*
Percentage of assembly mapped to chromosomes	99.78%	*≥ 95%*
Sex chromosomes	Z chromosome	*localised homologous pairs*
Organelles	Mitochondrial genome assembled	*complete single alleles*
Raw data accessions		
PacificBiosciences SEQUEL II	ERR10802375	
Hi-C Illumina	ERR10786005	
PolyA RNA-Seq Illumina	ERR12035175	
Genome assembly		
Assembly accession	GCA_949358125.1	
*Accession of alternate haplotype*	GCA_949358575.1	
Span (Mb)	422.6	
Number of contigs	68	
Contig N50 length (Mb)	13.2	
Number of scaffolds	35	
Scaffold N50 length (Mb)	25.4	
Longest scaffold (Mb)	31.7	
Genome annotation		
Number of protein-coding genes	18,351	
Number of gene transcripts	18,536	

* Assembly metric benchmarks are adapted from column VGP-2020 of “Table 1: Proposed standards and metrics for defining genome assembly quality” from ( Rhie *et al.*, 2021). ** BUSCO scores based on the lepidoptera_odb10 BUSCO set using v5.3.2. C = complete [S = single copy, D = duplicated], F = fragmented, M = missing, n = number of orthologues in comparison. A full set of BUSCO scores is available at https://blobtoolkit.genomehubs.org/view/ilIdaDimi2.1/dataset/CASHTA01/busco.

**Table 2.** Chromosomal pseudomolecules in the genome assembly of *Idaea dimidiata*, ilIdaDimi2.

INSDC accession	Chromosome	Length (Mb)	GC%
OX442326.1	1	31.73	35.5
OX442327.1	2	29.29	36.0
OX442328.1	3	28.94	35.5
OX442329.1	4	27.93	35.5
OX442331.1	5	27.79	36.0
OX442332.1	6	26.28	36.0
OX442333.1	7	25.42	36.0
OX442334.1	8	24.78	36.0
OX442335.1	9	23.67	36.0
OX442336.1	10	19.47	36.0
OX442337.1	11	18.5	36.5
OX442338.1	12	18.46	36.5
OX442339.1	13	18.25	36.0
OX442340.1	14	16.91	36.0
OX442341.1	15	15.17	36.5
OX442342.1	16	12.14	37.0
OX442343.1	17	11.51	36.0
OX442344.1	18	8.98	37.0
OX442345.1	19	8.66	37.5
OX442330.1	Z	27.8	36.0
OX442346.1	MT	0.02	18.0

The estimated Quality Value (QV) of the final assembly is 66.5 with *k*-mer completeness of 100%, and the assembly has a BUSCO v5.3.2 completeness of 98.3% (single = 97.8%, duplicated = 0.4%), using the lepidoptera_odb10 reference set ( *n* = 5,286).

Metadata for specimens, spectral estimates, sequencing runs, contaminants and pre-curation assembly statistics can be found at https://links.tol.sanger.ac.uk/species/572776.

## Genome annotation report

The *Idaea dimidiata* genome assembly (GCA_949358125.1) was annotated using the Ensembl rapid annotation pipeline ( Table 1; https://rapid.ensembl.org/Idaea_dimidiata_GCA_949358125.1/Info/Index). The resulting annotation includes 18,536 transcribed mRNAs from 18,351 protein-coding genes.

## Methods

### Sample acquisition and nucleic acid extraction

A male *Idaea dimidiata* (specimen ID NHMUK013805885, individual ilIdaDimi2) was collected from Hartslock Nature Reserve, Oxfordshire, UK (latitude 51.51, longitude –1.11) on 2021-07-29 using a light trap. The specimen was collected by Ian Sims (British Entomological and Natural History Society) and identified by David Lees (Natural History Museum), and then dry frozen at –80°C.

DNA was extracted at the Tree of Life laboratory, Wellcome Sanger Institute (WSI). The ilIdaDimi2 sample was weighed and dissected on dry ice with tissue set aside for Hi-C sequencing. Head and thorax tissue was disrupted using a Nippi Powermasher fitted with a BioMasher pestle. High molecular weight (HMW) DNA was extracted using the Qiagen MagAttract HMW DNA extraction kit. HMW DNA was sheared into an average fragment size of 12–20 kb in a Megaruptor 3 system with speed setting 30. Sheared DNA was purified by solid-phase reversible immobilisation using AMPure PB beads with a 1.8X ratio of beads to sample to remove the shorter fragments and concentrate the DNA sample. The concentration of the sheared and purified DNA was assessed using a Nanodrop spectrophotometer and Qubit Fluorometer and Qubit dsDNA High Sensitivity Assay kit. Fragment size distribution was evaluated by running the sample on the FemtoPulse system.

RNA was extracted from abdomen tissue of ilIdaDimi2 in the Tree of Life Laboratory at the WSI using TRIzol, according to the manufacturer’s instructions. RNA was then eluted in 50 μl RNAse-free water and its concentration assessed using a Nanodrop spectrophotometer and Qubit Fluorometer using the Qubit RNA Broad-Range (BR) Assay kit. Analysis of the integrity of the RNA was done using Agilent RNA 6000 Pico Kit and Eukaryotic Total RNA assay.

### Sequencing

Pacific Biosciences HiFi circular consensus DNA sequencing libraries were constructed according to the manufacturers’ instructions. Poly(A) RNA-Seq libraries were constructed using the NEB Ultra II RNA Library Prep kit. DNA and RNA sequencing was performed by the Scientific Operations core at the WSI on Pacific Biosciences SEQUEL II (HiFi) and Illumina NovaSeq 6000 (RNA-Seq) instruments. Hi-C data were also generated from remaining head and thorax tissue of ilIdaDimi2 using the Arima2 kit and sequenced on the Illumina NovaSeq 6000 instrument.

### Genome assembly, curation and evaluation

Assembly was carried out with Hifiasm ( Cheng *et al.*, 2021) and haplotypic duplication was identified and removed with purge_dups ( Guan *et al.*, 2020). The assembly was then scaffolded with Hi-C data ( Rao *et al.*, 2014) using YaHS ( Zhou *et al.*, 2023). The assembly was checked for contamination and corrected as described previously ( Howe *et al.*, 2021). Manual curation was performed using HiGlass ( Kerpedjiev *et al.*, 2018) and Pretext ( Harry, 2022). The mitochondrial genome was assembled using MitoHiFi ( Uliano-Silva *et al.*, 2023), which runs MitoFinder ( Allio *et al.*, 2020) or MITOS ( Bernt *et al.*, 2013) and uses these annotations to select the final mitochondrial contig and to ensure the general quality of the sequence.

A Hi-C map for the final assembly was produced using bwa-mem2 ( Vasimuddin *et al.*, 2019) in the Cooler file format ( Abdennur & Mirny, 2020). To assess the assembly metrics, the *k*-mer completeness and QV consensus quality values were calculated in Merqury ( Rhie *et al.*, 2020). This work was done using Nextflow ( Di Tommaso *et al.*, 2017) DSL2 pipelines “sanger-tol/readmapping” ( Surana *et al.*, 2023a) and “sanger-tol/genomenote” ( Surana *et al.*, 2023b). The genome was analysed within the BlobToolKit environment ( Challis *et al.*, 2020) and BUSCO scores ( Manni *et al.*, 2021; Simão *et al.*, 2015) were calculated.

Table 3 contains a list of relevant software tool versions and sources.

**Table 3.** Software tools: versions and sources.

Software tool	Version	Source
BlobToolKit	4.1.7	https://github.com/blobtoolkit/blobtoolkit
BUSCO	5.3.2	https://gitlab.com/ezlab/busco
Hifiasm	0.16.1-r375	https://github.com/chhylp123/hifiasm
HiGlass	1.11.6	https://github.com/higlass/higlass
Merqury	MerquryFK	https://github.com/thegenemyers/MERQURY.FK
MitoHiFi	2	https://github.com/marcelauliano/MitoHiFi
PretextView	0.2	https://github.com/wtsi-hpag/PretextView
purge_dups	1.2.3	https://github.com/dfguan/purge_dups
sanger-tol/ genomenote	v1.0	https://github.com/sanger-tol/genomenote
sanger-tol/ readmapping	1.1.0	https://github.com/sanger-tol/readmapping/tree/1.1.0
YaHS	1.2a	https://github.com/c-zhou/yahs

### Genome annotation

The BRAKER2 pipeline ( Brůna *et al.*, 2021) was used in the default protein mode to generate annotation for the *Idaea dimidiata* assembly (GCA_949358125.1) in Ensembl Rapid Release.

### Wellcome Sanger Institute – Legal and Governance

The materials that have contributed to this genome note have been supplied by a Darwin Tree of Life Partner. The submission of materials by a Darwin Tree of Life Partner is subject to the **‘Darwin Tree of Life Project Sampling Code of Practice’**, which can be found in full on the Darwin Tree of Life website here. By agreeing with and signing up to the Sampling Code of Practice, the Darwin Tree of Life Partner agrees they will meet the legal and ethical requirements and standards set out within this document in respect of all samples acquired for, and supplied to, the Darwin Tree of Life Project. 

Further, the Wellcome Sanger Institute employs a process whereby due diligence is carried out proportionate to the nature of the materials themselves, and the circumstances under which they have been/are to be collected and provided for use. The purpose of this is to address and mitigate any potential legal and/or ethical implications of receipt and use of the materials as part of the research project, and to ensure that in doing so we align with best practice wherever possible. The overarching areas of consideration are:

• Ethical review of provenance and sourcing of the material

• Legality of collection, transfer and use (national and international) 

Each transfer of samples is further undertaken according to a Research Collaboration Agreement or Material Transfer Agreement entered into by the Darwin Tree of Life Partner, Genome Research Limited (operating as the Wellcome Sanger Institute), and in some circumstances other Darwin Tree of Life collaborators.

## Data Availability

European Nucleotide Archive: *Idaea dimidiata* (single dot wave). Accession number PRJEB58928; https://identifiers.org/ena.embl/PRJEB58928. ( Wellcome Sanger Institute, 2023) The genome sequence is released openly for reuse. The *Idaea dimidiata* genome sequencing initiative is part of the Darwin Tree of Life (DToL) project. All raw sequence data and the assembly have been deposited in INSDC databases. Raw data and assembly accession identifiers are reported in Table 1.

## Author information

Members of the Natural History Museum Genome Acquisition Lab are listed here: https://doi.org/10.5281/zenodo.4790042.

Members of the Darwin Tree of Life Barcoding collective are listed here: https://doi.org/10.5281/zenodo.4893703.

Members of the Wellcome Sanger Institute Tree of Life programme are listed here: https://doi.org/10.5281/zenodo.4783585.

Members of Wellcome Sanger Institute Scientific Operations: DNA Pipelines collective are listed here: https://doi.org/10.5281/zenodo.4790455.

Members of the Tree of Life Core Informatics collective are listed here: https://doi.org/10.5281/zenodo.5013541.

Members of the Darwin Tree of Life Consortium are listed here: https://doi.org/10.5281/zenodo.4783558.
