# Supplementary material for: The genome sequence of the Single-dotted Wave, Idaea dimidiata (Hufnagel, 1767)
Source: Wellcome Open Res. 2023 Dec 5;8:557. [Version 1] doi: 10.12688/wellcomeopenres.20185.1 (PMC12476536; doi:10.12688/wellcomeopenres.20185.1)
Supplement: Peer review history

# Reviewer report: Reviewer response for version 1

**DOI:** 10.21956/wellcomeopenres.22348.r131469

**Published:** 2025-09-27

**Authors:** Jose Martinez

**Affiliations:** 1 University of Florida, Florida, USA

Sims et al. present a genome assembly of the The Single-dotted Wave, *Idaea dimidiata* (Hufnagel, 1767), which demonstrates a high degree of completeness, as evidenced by the elevated BUSCO score. The assembly is organized into 20 chromosomal pseudomolecules, including the assembled Z sex chromosome. This, combined with other well-described methodologies, has produced high-quality genomic data.

 The dataset is built on a solid and conventional rationale, offering clear, comprehensible descriptions that make it easy to navigate. The protocols are suitable, and the accompanying materials are documented in a replicable format. Its strong standardization, combined with thorough illustrations and open accessibility, positions it as a highly valuable tool for multiple scientific and practical applications. In addition, the quality of the genome may be further refined through manual curation, thereby enhancing its utility as a reference for examining the systematics and evolutionary relationships within Geometridae. It also offers a valuable framework for the annotation of genes of particular relevance to these moth lineages.

 Congratulations!

Are sufficient details of methods and materials provided to allow replication by others?

Yes

Is the rationale for creating the dataset(s) clearly described?

Yes

Are the datasets clearly presented in a useable and accessible format?

Yes

Are the protocols appropriate and is the work technically sound?

Yes

Reviewer Expertise:

Evolutionary Biology, Ecology, IPM, Integrative Biology, Entomology, Genomics

I confirm that I have read this submission and believe that I have an appropriate level of expertise to confirm that it is of an acceptable scientific standard.

# Reviewer report: Reviewer response for version 1

**DOI:** 10.21956/wellcomeopenres.22348.r131473

**Published:** 2025-09-18

**Authors:** Taslima Sheikh

**Affiliations:** 1 Baba Ghulam Shah Badshah University, Rajouri, India

The manuscript presents a scientifically sound and valuable genome assembly of *Idaea dimidiata*, including the mitochondrial genome and comprehensive gene annotation.

 The study is well prepared, with clear results that will serve as an important resource for Lepidoptera research.

 Overall, it is a strong and impactful contribution. Congratulations for this wonderful work.

Are sufficient details of methods and materials provided to allow replication by others?

Yes

Is the rationale for creating the dataset(s) clearly described?

Yes

Are the datasets clearly presented in a useable and accessible format?

Yes

Are the protocols appropriate and is the work technically sound?

Yes

Reviewer Expertise:

lepidoptera

I confirm that I have read this submission and believe that I have an appropriate level of expertise to confirm that it is of an acceptable scientific standard.
